# Adherence to the Mediterranean Diet: A Tool to the Therapeutic Approaches for Patients With Temporomandibular Joint Disorders

**DOI:** 10.7759/cureus.68698

**Published:** 2024-09-05

**Authors:** Lydia Kesidou, Theodore Fasilis, Athanasia Alexoudi, Olga Kesidou, Georgios Tsatov, Areti Tsoga

**Affiliations:** 1 Department of Dentistry, Santorini General Hospital, Santorini, GRC; 2 1st Department of Neurosurgery/Clinical Neuropsychology Laboratory, School of Medicine, Faculty of Health Sciences, National and Kapodistrian University of Athens, Athens, GRC; 3 Department of Neurosurgery, National and Kapodistrian University of Athens, Evangelismos Hospital, Athens, GRC; 4 Department of Neurology, Neurological Institute of Athens, Athens, GRC; 5 Department of Otolaryngology-Head and Neck Surgery, Athens Medical Group/Iatriko Psichikou, Athens, GRC; 6 Department of Dentistry, Private Practice, Athens, GRC; 7 Department of Public Health Policy, University of West Attica, Athens, GRC

**Keywords:** gut-brain axis, joint osteoarthritis, mediterranean diet, myofascial pain, neuromuscular junction disorders, nutrition, physical therapy, public heath dentist, temporomandibular disorders, tmj disorders and dentistry

## Abstract

Background and aim: Temporomandibular joint disorder (TMD) is characterized by symptoms such as clenching, clicking, and locking of the jaw, often due to improper positioning affecting occlusion. Nearly half of TMD patients rarely require treatment, as symptoms typically diminish on their own within a year. Nevertheless, a significant majority of persons who are diagnosed with TMD do necessitate therapy, and it may take up to three years for complete remission to occur. This study aims to determine the extent to which a healthy nutritional model, specifically the Mediterranean diet, can enhance the effectiveness of existing therapeutic treatments, like physiotherapy with warm pads.

Methods: An interventional study design was implemented. Baseline scores were obtained pre- and post-intervention, while Mediterranean diet adherence was evaluated once at the beginning. A dependent samples t-test and a one-way multivariate analysis of covariance (MANCOVA) were used to test the experimental hypotheses.

Results: There is a statistically significant difference (p=0.04) between the three groups associated with Mediterranean diet adherence, as indicated by the mean differences on the Jaw Functional Limitation Scale (JFLS-20) questionnaire. Participants following a medium or high level of Mediterranean diet (≥18) reported fewer problems with jaw functionality both before and after the intervention compared to those with low (<18) adherence to the diet.

Conclusion: Adherence to the Mediterranean diet appears to have a therapeutic effect on patients with TMD, offering a new dimension to their treatment. The primary benefit is the low cost of treatment, as the diet is easily accessible. This dietary approach could significantly enhance the management of TMD symptoms.

## Introduction

Musculoskeletal conditions are of great importance both individually and at a societal level and appear to be closely associated not only with decreasing life expectancy but also with increasing longevity [1]. Temporomandibular joint disorders (TMDs) are a term used to describe a group of dysfunctions involving the masseter muscles and temporomandibular joint (TMJ) [2].

TMJ pain has been reported to affect approximately 10% of the adult population and is considered to be the most common chronic orofacial pain condition. The terms "TMDs" and "TMJ disorders" are used to describe a group of musculoskeletal disorders that occur in the TMJ region [1]. They are characterized by pain in the muscles of mastication, the TMJ, or both and potentially other orofacial muscles. In addition to constant pain, pain during palpation and pain during function such as speaking and swallowing have also been reported [3]. It is the most common cause of pain of non-odontogenic origin in the oromandibular region and the second most common musculoskeletal condition (after chronic low back pain) which is associated with pain and disablement to the degree that it interferes with a person's daily activities, psychosocial functioning, and quality of life [4]. Symptoms include masseter myalgia, pain in preauricular regions of interest, headaches, limited mouth opening, deviation of the jaw during opening, as well as clicking, grinding, clenching, and locking of the mandible [5].

The prevalence of TMD depends largely on the symptoms and their occurrence in terms of frequency and duration, ranging from 12% to 42.7% in the adult population, with 3-9% needing treatment [2]. The prevalence increases to 50% in the adult population when all symptoms associated with TMD are included [6].

The diagnosis of TMD should be based on a combination of clinical examination and the reported medical history of the patient [7]. There are diagnostic criteria for TMDs, while further tools are constantly being added [8], since the process of diagnosis is extremely complex and multifactorial because of the multiple disciplines associated with the head [9]. The Research Diagnostic Criteria for Temporomandibular Disorders (RDC/TMDs) represents a highly sophisticated and valuable instrument that provides diagnosis based on clinical criteria for the accurate assessment of TMD in both pediatric and adult populations [10]. The RDC/TMDs were gradually replaced by the updated Diagnostic Criteria for TMD (DC-TMD) [4].

The initial approach for the treatment of TMD is to decrease pain and disability, which can be achieved through a variety of interventions and a multidisciplinary approach. Conservative interventions include patient education, physiotherapy, psychosocial management, and splints. The benefits of thermotherapy include pain relief, reduction in tension, and increased muscle extensibility due to collagen stretching, which consequently improves mouth opening and jaw functions [11]. A combination of these approaches is common in clinical practice [12]. In general, the pharmacological approach, alone or in combination with other approaches, is still the main clinical approach [13]. Nevertheless, the efficacy of available drugs for TMD is questionable, and their side effects may limit their clinical use [14]. Therefore, other alternatives, such as natural products, have been explored. Natural products can be mixtures of substances from plants and animals or even isolated compounds. Traditionally, they have been an important source of compounds with therapeutic potential for the treatment of many diseases, including inflammatory and painful conditions [15]. Few clinical trials have evaluated natural products in patients with TMD. For example, a preliminary study showed that avocado and soy extract reduced pain and improved quality of life in patients with TMJ degeneration [16]. In addition, grape seed extract has been suggested as a promising anti-inflammatory agent [17], particularly due to its anthocyanin content, which is considered to be part of a group of compounds that may reduce neuroinflammation [18].

The Mediterranean diet refers not only to the type of food consumed but also to the lifestyle and social customs associated with the way individuals eat [19]. This diet, first described by Keys [20], is characterized by high consumption of fruits, vegetables, unrefined cereals, olive oil, nuts, and seafood; moderate consumption of chicken, dairy products, and red wine; and low consumption of red meat. Adherence to this diet is associated with a reduced risk of several chronic diseases, especially cardiovascular diseases, various types of cancer, and type 2 diabetes mellitus [21]. The Mediterranean diet can also play a protective role against psychological disorders due to its high content of fiber, omega-3 fatty acids, vitamins B and E, magnesium, antioxidants, and phytoestrogens [22]. Moreover, several important components in this diet such as legumes, nuts, and fish are important factors in controlling and reducing the process of nerve damage [19].

The aim of the present study is to investigate the effect of a healthy nutritional pattern, in the form of a Mediterranean diet, on the effectiveness of the external thermal application in the treatment of TMD, as measured by two questionnaires, the main benefit being the reduction in the financial burden of treatment due to specific dietary patterns.

## Materials and methods

Hypotheses

Overall, we have three hypotheses. The first two hypotheses support that there will be a statistically significant difference in the mean scores of the two questionnaires associated with pain (H1) and jaw functionality (H2), before and after the intervention (thermal/heating pads). The third hypothesis (H3) states that there will be a statistically significant difference as anticipated among the three groups categorized by adherence to the Mediterranean diet, with respect to the mean changes in scores on the two aforementioned questionnaires of pain and jaw functionality, after controlling for age and gender.

Study design

Accordingly, we implemented an interventional study design. Despite the already proven validity of the therapeutic approach used [11], we were mainly interested in the impact of the diet on the specific intervention. Raw data were obtained in the form of self-referred questionnaires associated with pain, jaw functionality, and Mediterranean diet preference.

This study was approved by the Ethics and Research Committee of Santorini Hospital (approval number: 3625/13-06-2023). All participants were provided with comprehensive information regarding the study and subsequently granted their informed consent by signing the requisite documentation.

Participants and eligibility criteria

In total, 16 individuals participated in the present study (six males and 10 females), with a mean age of 41.9 years old (SD=12.324). Participants were recruited through referrals to the Dental Department of the General Hospital of Thira in Santorini, Greece, as part of their routine medical evaluations. All TMD patients had received an RDC/TMD pain diagnosis.

In order to ensure the validity of the study, participants with coexistence of other neurodegenerative diseases and/or psychiatric comorbidity have been excluded from the study.

Data collection

Individuals were administered three questionnaires. The first open-access questionnaire is the Temporomandibular Disorder Pain Screener Questionnaire (TMD-PAIN SCREENER), the second is the Jaw Functional Limitation Scale (JFLS-20), and the third is the Mediterranean Diet Scale (MDS); the questionnaire was permitted by Prof. D.B. Panagiotakos. The MDS was administered once, while the rest two questionnaires (TMD-PAIN SCREENER and JFLS-20) were administered pre- and post-intervention.

The MDS is a questionnaire about the Mediterranean diet. In specific, it is useful in assessing the nutritional status of an individual and investigating the relationship of the Mediterranean diet with various health outcomes. The objective is the degree of recognition of the Mediterranean Standard, including 11 different factors that are calibrated using functions that are weighted from 0 to 5 and score values from 0 to 55 [23].

The JFLS-20 is a scale about the functional limitation of the masticatory system. It is a self-assessment questionnaire, consisting of 20 items associated with jaw functionality [24].

The TMD-PAIN SCREENER is an applicable questionnaire for screening the adult population in order to recognize patients in need of further TMD examination and management [25].

Data collection procedure

After the diagnosis of RDC/TMD, participants were evaluated according to the hospital's protocol. Subsequently, all participants received thermal therapy using Nexcare mini heating gel pads (manufactured by Nexcare) in addition to standard care. The heating pads were applied to the TMJ area for 15 minutes. The temperature setting was maintained at approximately 35° throughout the study. Upon completion of the therapy, all participants returned to the hospital for reevaluation.

Statistical analysis

A dependent samples t-test was performed based on the comparison of the mean scores in the questionnaires given pre- and post-intervention. In specific, we manipulated as independent variable (IV) the intervention, with two levels of measurement (pre- and post-intervention), and as dependent variables (DVs) the scores from the questionnaires employed before and after the intervention.

In addition, we calculated the mean difference between pre- and post-intervention, and we conducted a one-way multivariate analysis of covariance (MANCOVA), in order to examine the relationship between the Mediterranean diet and its impact on the intervention based on the two questionnaires after controlling for gender and age. MDS scores were categorized into three levels (low (>18), medium (18-36), and high (>36)) and were used as an IV. The difference of before and after scores in the questionnaires was used as our DV. 

The normality of distribution of the DVs on the levels of the IV was assessed with the Shapiro-Wilk test of normality. According to the Shapiro-Wilk test, the results indicate that both JFLS-20 and TMD-PAIN SCREENER are normally distributed pre-intervention (p=0.984, p=0.146) and post-intervention (p=0.684, p=0.636), respectively. Data were analyzed with the use of IBM SPSS Statistics for Windows, Version 24.0 (Released 2016; IBM Corp., Armonk, New York, United States). The significance level was set at a=0.05 (CI 95%).

## Results

The participants were almost the same percentage, females and males in their 40s, with a university degree and normal BMI, as shown in Table 1.

**Table 1:** Descriptive statistics concerning demographic data.

Demographics	Participants (N=16)		
	Male	Female	Total
Age	46.41 (±8.553)	45.02 (±11.002)	41.90 (±11.193)
Years of education	16.02 (±2.236)	16.17 (±2.470)	16.25 (±2.272)
Height	1.80 (±.098)	1.63 (±.040)	1.70 (±.107)
Weight	75.50 (±7.141)	67.00 (±9.592)	70.40 (±9.348)
BMI	23.46 (±3.398)	25.14 (±3.865)	24.47 (±3.591)

Seventy percent of the participants had no medical problem, while 20% reported arterial pressure complications and 10% diabetes mellitus. Furthermore, 70% of the participants reported an average healthy diet, 20% reported a very healthy diet, and only 10% reported a very unhealthy diet. Only two participants answered that they do not consume fast food at all. Nine participants reported an average consumption of 1-4 times per month, and five participants reported at least two times per week, with one participant consuming fast food more than three times per week. In addition, 60% of the participants reported that they never do physical exercise, and 40% reported training 3-5 times per week. Moreover, 70% stated that they were non-smokers, and 30% were smokers. Lastly, 90% of the participants reported that they do not consume alcohol, and only 10% consume systematically alcohol. None of the participants reported any drug use.

The paired samples t-test demonstrated that participants had statistically significantly lower scores after the intervention in JFLS-20 (t(11)=5.710, p<0.001). JFLS-20 scores following the intervention decreased from 61.67 (±32.740) to 37.83 (±20.997), an improvement of 23.84 (±14.459), with a large effect size (Table 2). There was also a statistically significant difference in TMD-PAIN SCREENER scores after the intervention (t(11)=2.755, p=0.019). The mean scores demonstrated an improvement in the questionnaire's scores pre- and post-intervention, from 4.50 (±1.679) to 3.33 (±1.557), an improvement of 1.17 (±1.467), with a medium effect size (Table 2).

**Table 2:** Inferential statistics (dependent samples t-test) concerning the scores in the questionnaires JFLS-20 and TMD-PAIN SCREENER pre- and post-intervention. JFLS-20: Jaw Functional Limitation Scale; TMD-PAIN SCREENER: Temporomandibular Disorder Pain Screener Questionnaire

	Intervention				N=16		
Questionnaires	Pre		Post		t	p	Cohen's d
	M	SD	M	SD			
JFLS-20	61.67	32.740	37.83	20.997	5.710	0.000	1.29
TMD-PAIN SCREENER	4.50	1.679	3.33	1.557	2.755	0.019	0.79

The one-way MANCOVA revealed a statistically significant difference among the groups on the composite of dependent variables when controlling for the covariates of age and gender F(4, 20)=2.899, p<0.05, Wilks' Λ=0.401, partial η2=0.367, with a medium effect size, as illustrated in Table 3.

**Table 3:** MANCOVA concerning the levels of the Mediterranean diet (MDS-11) and the difference in the scores pre- and post-intervention of JFLS-20 and TMD-PAIN SCREENER, after controlling for gender and age. MANCOVA: multivariate analysis of covariance; JFLS-20: Jaw Functional Limitation Scale; TMD-PAIN SCREENER: Temporomandibular Disorder Pain Screener Questionnaire

	M (SD)	f	p	Wilks' Λ	Partial η^2^
MDS-11	31.06 (5.434)	2.899	0.048	0.401	0.367

These findings support our hypotheses (H1, H2, H3), indicating that adherence to a Mediterranean diet is associated with improved TMJ functionality and reduced perceived pain levels. Participants who maintained moderate to high adherence to the Mediterranean dietary pattern demonstrated significantly lower scores on JFLS-20 and TMD scales post-intervention, compared to those with low adherence to the Mediterranean diet as shown in Table 4.

**Table 4:** Percentage of participants (%) who showed improvement in JFLS-20 and TMD-PAIN SCREENER questionnaires according to the levels of the Mediterranean diet. JFLS-20: Jaw Functional Limitation Scale; TMD-PAIN SCREENER: Temporomandibular Disorder Pain Screener Questionnaire

	Mediterranean diet (MDS-11)		
Questionnaires	Low (N=2)	Medium (N=11)	High (N=3)
	N (%)	N (%)	N (%)
JFLS-20	2 (12.5%)	10 (62.5%)	3 (18.75%)
TMD-PAIN SCREENER	0 (0%)	9 (56.25%)	3 (18.75%)

## Discussion

The present study aimed to investigate the association between adherence to the Mediterranean diet and the risk of developing chronic pain in the temporomandibular area and especially joint. Our findings suggest a significant inverse relationship between higher adherence to the Mediterranean dietary pattern and the incidence of TMDs.

These results align with previous epidemiological studies that have linked the Mediterranean diet to a reduced risk of chronic pain as well as orofacial pain [26]. The protective effects of this dietary pattern may be attributed to the synergistic action of its nutrient-rich components, including monounsaturated fatty acids, antioxidants, and anti-inflammatory compounds [27].

Notably, our analysis revealed that the strongest adherence to the Mediterranean diet was associated with a substantially lower risk of jaw joint functionality and pain (osteoarthritis), corroborating the findings from the literature [28]. According to a systematic review and meta-analysis, consuming a diet rich in antioxidants and anti-inflammatory compounds could have positive health effects [29]. These substances, found in various foods, may help protect cells from damage and reduce harmful inflammation in the body.

The connection could be attributed to certain elements of the Mediterranean diet particularly olive oil, fish, and vegetables, which have neuroprotective and antioxidant qualities. These components have demonstrated the abilities to counteract oxidative stress and reduce inflammation in the nervous system. This aligns with the processes involved in central sensitization by two significant factors, firstly N-methyl-D-aspartate receptor (NMDA) and gamma-aminobutyric acid (GABA) activation and secondly glycine receptor inhibition, which are presumed to be present in most chronic pain conditions [30].

While our results show statistically significant improvements in JFLS-20 and TMD-PAIN SCREENER scores, it's important to consider their clinical relevance. Our observed improvements in JFLS-20 and in TMD-PAIN SCREENER exceed these thresholds, suggesting that adherence to the Mediterranean diet may lead to clinically meaningful benefits for TMD patients.

Due to the scarcity of relevant literature in the field, we were unable to identify comparable studies. Consequently, a comparative analysis between our findings and those of other researches could not be conducted.

Limitations

However, some limitations should be acknowledged. Firstly, dietary intake was self-reported, which may be subject to recall bias and measurement errors. Secondly, our study did not account for potential changes in dietary patterns over time, which could have influenced the observed associations. Additionally, residual confounding from unmeasured factors, such as genetic predisposition or environmental exposures, cannot be ruled out.

To investigate the notion of a potential influence of the Mediterranean diet, it would be necessary to extend the duration of the study.

One further constraint is that the medical conditions are based on self-reports, potentially leading to bias.

A notable methodological constraint of this study lies in our inability to account for the potential impact of biochemical markers on the observed relationship between adherence to the Mediterranean diet and the measured outcomes. The absence of these markers in our analysis represents a significant limitation, as they may play a crucial role in elucidating the underlying mechanisms of the observed associations.

The presence of residual confounding factors, such as socioeconomic status, remains a significant limitation in this study. Moreover, the potential for reverse causality cannot be discounted; specifically, there exists the plausible scenario wherein individuals experiencing a higher quality of life may, as a consequence, adopt superior dietary habits.

To support our results, a large number of patients should be included, in particular for the high adherence group. Additionally, a longitudinal observational study and a dietary intervention study are necessary to further explore the impact of the Mediterranean diet on TMD interventions. Furthermore, more cohort studies are needed to assess the role of this specific diet.

Despite these limitations, our findings contribute to the growing body of evidence supporting the beneficial effects of the Mediterranean diet on TMDs. Our study included a comprehensive assessment of dietary habits using validated food frequency questionnaires. Given the substantial burden of TMDs and the lack of effective treatments, adopting a Mediterranean-style dietary pattern may represent a promising strategy for disease prevention and management.

Future studies

Similar studies should be conducted in other geographic regions and ethnic groups before making broad recommendations for implementation in these populations. To be confident in the results, future studies in other dietary cultures that consider accurate results such as a radiographic test or biochemical markers are needed to trust the results. Caution should be exercised when extending the results presented here to larger contexts, as the data were collected on a Greek island with a Mediterranean tradition and culture. While more study is needed, incorporating such nutrient-dense foods into one's diet might contribute to better overall health and potentially lower the risk of certain diseases. Future research should further elucidate the specific components and mechanisms underlying the protective effects of the Mediterranean diet. Additionally, large-scale interventional studies are warranted to establish causality and evaluate the potential of the Mediterranean diet as a therapeutic approach for TMDs.

## Conclusions

This study revealed a significant inverse correlation between Mediterranean diet adherence and TMD prevalence. Higher adherence was associated with lower jaw functional limitation scores. The Mediterranean dietary pattern demonstrates potential as a cost-effective and widely implementable intervention for mitigating the prevalence and progression of TMDs, suggesting substantial public health implications for both the prevention and management of this condition. The study supports the potential of nutritional interventions in TMD treatment and underscores the importance of healthy dietary habits. The results confirm both hypotheses, demonstrating that the intervention significantly reduced TMD-related pain and improved jaw functional limitations, as assessed by self-reported measures.

## References

[1] LeResche L (1997). Epidemiology of temporomandibular disorders: implications for the investigation of etiologic factors. Crit Rev Oral Biol Med.

[2] Badel T, Lovko SK, Zadravec D (2014). Anxiety and temporomandibular disorders: a relationship in chronic pain development. Anxiety Disorders: Risk Factors, Genetic Determinants and Cognitive-Behavioral Treatment.

[3] Truelove EL, Sommers EE, LeResche L, Dworkin SF, Von Korff M (1992). Clinical diagnostic criteria for TMD. New classification permits multiple diagnoses. J Am Dent Assoc.

[4] Schiffman E, Ohrbach R, Truelove E (2014). Diagnostic criteria for temporomandibular disorders (DC/TMD) for clinical and research applications: recommendations of the International RDC/TMD Consortium Network and Orofacial Pain Special Interest Group. J Oral Facial Pain Headache.

[5] Collins T (2020). Temporomandibular joint disorders. InnovAiT.

[6] Lövgren A, Visscher CM, Häggman-Henrikson B, Lobbezoo F, Marklund S, Wänman A (2016). Validity of three screening questions (3Q/TMD) in relation to the DC/TMD. J Oral Rehabil.

[7] Berni KC, Dibai-Filho AV, Pires PF, Rodrigues-Bigaton D (2015). Accuracy of the surface electromyography RMS processing for the diagnosis of myogenous temporomandibular disorder. J Electromyogr Kinesiol.

[8] Gonzalez YM, Greene CS, Mohl ND (2008). Technological devices in the diagnosis of temporomandibular disorders. Oral Maxillofac Surg Clin North Am.

[9] Medlicott MS, Harris SR (2006). A systematic review of the effectiveness of exercise, manual therapy, electrotherapy, relaxation training, and biofeedback in the management of temporomandibular disorder. Phys Ther.

[10] Szyszka-Sommerfeld L, Sycińska-Dziarnowska M, Budzyńska A, Woźniak K (2022). Accuracy of surface electromyography in the diagnosis of pain-related temporomandibular disorders in children with awake bruxism. J Clin Med.

[11] Furlan RM, Giovanardi RS, Britto AT, Oliveira e Britto DB (2015). The use of superficial heat for treatment of temporomandibular disorders: an integrative review. Codas.

[12] (2019). Temporomandibular disorders: improving outcomes using a multidisciplinary approach [erratum]. J Multidiscip Healthc.

[13] Christidis N, Al-Moraissi EA, Al-Ak'hali MS (2024). Psychological treatments for temporomandibular disorder pain-a systematic review. J Oral Rehabil.

[14] Oliveira JP, Nampo FK, Souza MT, Cercato LM, Camargo EA (2020). The effect of natural products in animal models of temporomandibular disorders. J Appl Oral Sci.

[15] Chopra B, Dhingra AK (2021). Natural products: a lead for drug discovery and development. Phytother Res.

[16] Catunda IS, Vasconcelos BC, Andrade ES, Costa DF (2016). Clinical effects of an avocado-soybean unsaponifiable extract on arthralgia and osteoarthritis of the temporomandibular joint: preliminary study. Int J Oral Maxillofac Surg.

[17] Baydar NG, Özkan G, Yaşar S (2007). Evaluation of the antiradical and antioxidant potential of grape extracts. Food Cont.

[18] Carvalho FB, Gutierres JM, Bueno A (2017). Anthocyanins control neuroinflammation and consequent memory dysfunction in mice exposed to lipopolysaccharide. Mol Neurobiol.

[19] Hernández-Galiot A, Goñi I (2017). Adherence to the Mediterranean diet pattern, cognitive status and depressive symptoms in an elderly non-institutionalized population. Nutr Hosp.

[20] Keys A (1995). Mediterranean diet and public health: personal reflections. Am J Clin Nutr.

[21] Mantzorou M, Vadikolias K, Pavlidou E, Tryfonos C, Vasios G, Serdari A, Giaginis C (2021). Mediterranean diet adherence is associated with better cognitive status and less depressive symptoms in a Greek elderly population. Aging Clin Exp Res.

[22] Sadeghi O, Keshteli AH, Afshar H, Esmaillzadeh A, Adibi P (2021). Adherence to Mediterranean dietary pattern is inversely associated with depression, anxiety and psychological distress. Nutr Neurosci.

[23] Panagiotakos DB, Pitsavos C, Stefanadis C (2006). Dietary patterns: a Mediterranean diet score and its relation to clinical and biological markers of cardiovascular disease risk. Nutr Metab Cardiovasc Dis.

[24] Ohrbach R, Larsson P, List T (2008). The jaw functional limitation scale: development, reliability, and validity of 8-item and 20-item versions. J Orofac Pain.

[25] Gonzalez YM, Schiffman E, Gordon SM, Seago B, Truelove EL, Slade G, Ohrbach R (2011). Development of a brief and effective temporomandibular disorder pain screening questionnaire: reliability and validity. J Am Dent Assoc.

[26] Kaushik AS, Strath LJ, Sorge RE (2020). Dietary interventions for treatment of chronic pain: oxidative stress and inflammation. Pain Ther.

[27] Camargo A, Delgado-Lista J, Garcia-Rios A (2012). Expression of proinflammatory, proatherogenic genes is reduced by the Mediterranean diet in elderly people. Br J Nutr.

[28] Morales-Ivorra I, Romera-Baures M, Roman-Viñas B, Serra-Majem L (2018). Osteoarthritis and the Mediterranean diet: a systematic review. Nutrients.

[29] Bloomfield HE, Koeller E, Greer N, MacDonald R, Kane R, Wilt TJ (2016). Effects on health outcomes of a Mediterranean diet with no restriction on fat intake: a systematic review and meta-analysis. Ann Intern Med.

[30] Costa YM, Conti PC, de Faria FA, Bonjardim LR (2017). Temporomandibular disorders and painful comorbidities: clinical association and underlying mechanisms. Oral Surg Oral Med Oral Pathol Oral Radiol.

